# A high-protein diet containing inulin/oligofructose supports body weight gain associated with lower energy expenditure and carbohydrate oxidation, and alters faecal microbiota in C57BL/6 mice

**DOI:** 10.1017/jns.2021.42

**Published:** 2021-07-13

**Authors:** Franziska Koch, Michael Derno, Martina Langhammer, Armin Tuchscherer, Harald M. Hammon, Manfred Mielenz, Cornelia C. Metges, Björn Kuhla

**Affiliations:** 1 Institute of Nutritional Physiology “Oskar Kellner”, Institute for Farm Animal Biology (FBN), Wilhelm-Stahl-Allee 2, 18196 Dummerstorf, Germany; 2 Institute of Genetics and Biometry, Service Group Lab Animal Facility, Institute for Farm Animal Biology (FBN), Wilhelm-Stahl-Allee 2, 18196 Dummerstorf, Germany; 3 Institute of Genetics and Biometry, Livestock Genetics and Breeding Unit, Institute for Farm Animal Biology (FBN), Wilhelm-Stahl Allee 2, 18196 Dummerstorf, Germany

**Keywords:** Energy expenditure, High-protein diet, Inulin, Microbiota, Nitrogen excretion, C, control, COX, carbohydrate oxidation, EB, energy balance, FOS, oligofructose, FOX, fat oxidation, HP, high protein, RQ, respiratory quotient, TEE, total energy expenditure, +I, inulin/oligofructose

## Abstract

Prebiotic supplements and high-protein (HP) diets reduce body weight and modulate intestinal microbiota. Our aim was to elucidate the combined effect of an inulin/oligofructose (FOS) and HP diet on body weight gain, energy metabolism and faecal microbiota. Forty male C57BL/6NCrl mice were fed a control (C) diet for 2 weeks and allocated to a C or HP (40 % protein) diet including no or 10 % inulin/FOS (C + I and HP + I) for 4 weeks. Inulin/FOS was added in place of starch and cellulose. Body weight, food intake, faecal energy and nitrogen were determined. Indirect calorimetry and faecal microbiota analysis were performed after 3 weeks on diets. Body weight gain of HP-fed mice was 36 % lower than HP + I- and C-fed mice (*P* < 0⋅05). Diet digestibility and food conversion efficiency were higher in HP + I- than HP-fed mice (*P* < 0⋅01), while food intake was comparable between groups. Total energy expenditure (heat production) was 25 % lower in HP + I- than in C-, HP- and C + I-fed mice (*P* < 0⋅001). Carbohydrate oxidation tended to be 24 % higher in HP- than in HP + I-fed mice (*P* < 0⋅05). Faecal nitrogen excretion was 31–45 % lower in C-, C + I- and HP + I- than in HP-fed mice (*P* < 0⋅05). Faecal *Bacteroides–Prevotella* DNA was 2⋅3-fold higher in C + I- and HP + I- relative to C-fed mice (*P* < 0⋅05), but *Clostridium leptum* DNA abundances was 79 % lower in HP + I- than in HP-fed mice (*P* < 0⋅05). We suggest that the higher conversion efficiency of dietary energy of HP + I but not C + I-fed mice is caused by higher digestibility and lower heat production, resulting in increased body mass.

## Introduction

Prebiotics and high-protein (HP) diets are considered as ‘functional foods’ with potential health benefits^(1)^. Prebiotics, such as inulin and oligofructose (FOS), are defined as non-digestible dietary fibre^(2)^ and are naturally present as plant storage carbohydrates in, e.g., chicory roots, onions or bananas^(3)^. In mice, inulin is fermented in the distal colon and FOS in the caecum and proximal colon^(4)^, inducing beneficial alterations of the gut microbiota^(5)^. Inulin and FOS promote the growth of *Lactobacilli* and *Bifidobacteria* in rodents^(6)^ and humans^(2)^, thereby improving the gut health of the host, e.g. by supporting the digestion and absorption of nutrients and minerals, inhibiting pathogenic bacteria or stimulating gastrointestinal immune functions^(2)^. Furthermore, FOS serves as an energy source for polysaccharide-cleaving bacteria, e.g. *Bacteroides*, which, however, require an additional nitrogen (N) source for protein synthesis^(2,7)^. Supplementing a diet for rodents with 7⋅5 % FOS or xylo-oligosaccharide (XOS) and gum arabic increased faecal N losses and reduced renal N excretion^(8)^. Furthermore, prebiotics increases gastric filling and intestinal motility and reduces body weight due to increased satiety in humans and rodents^(9–11)^. As a potential negative effect, ingestion of high amounts (>15 g/d) of inulin induces flatulence, abdominal pain and bloating in humans, which may limit the long-term application of inulin and FOS at high concentrations in the diet^(12,13)^.

Diets with a high protein (30–55 %) but low carbohydrate content promote body weight loss^(14)^ and increase muscle mass^(15)^, but also induce oxidative stress^(16)^. In mice, feeding a 60 % HP diet for 12 weeks reduced body weight by 21 % and fat mass by 39 % compared with a control diet containing 20 % protein^(17)^. After 8 weeks of ingestion of a 28 % protein-rich diet, non-obese humans reduced their body weight and fat mass by 3 and 9 %, respectively^(18)^. In overweight women, a 30 % HP-high-fibre diet, with fibres originating from oats, legumes, nuts, dried fruits, whole-grain breads and cereals (>35 g fibre/d), reduced body weight gain after 10 weeks compared with the control diet^(19)^. The reduction in body weight of non-obese rats fed with a 55 % HP diet might be linked to the inhibition of *de novo* lipogenesis limiting fat mass deposition^(20)^, but increasing thermogenesis and energy expenditure is another possibility^(21,22)^. The thermic effect of dietary protein is the highest compared to that of the other macronutrients^(22)^. In diets with the higher protein content (25–55 %), greater thermogenesis and energy expenditure were only found during the first 2–4 weeks of feeding, but not when HP diets were fed for 6 weeks and longer to rodents^(23,24)^ and humans^(25,26)^. Thus, thermogenesis seems to be stimulated shortly after the initiation of HP diet feeding. High-protein diets may also influence gut microbiota composition^(27,28)^. In the colon, undigested proteins are metabolised by the microbiota, predominantly involving proteolytic bacteria such as *Bacteriodes* in human faecal inoculates^(29)^. In rodents, feeding an HP diet (53 % protein) for 15 d compared to a control diet (14 % protein) reduced the abundance of *Clostridium coccoides* group and *Clostridium leptum* group in the large intestine^(30)^.

Taken together, consumption of an HP diet or inulin supplementation has been shown to reduce body weight and affect large intestinal microbiota. We hypothesised that consumption of an HP diet containing inulin/FOS has additive beneficial effects on body weight reduction involving mechanisms related to digestibility and energy metabolism. The objectives of the present study were to investigate the effects of inulin/FOS included in an HP (40 % protein) compared to a control (18 % protein) diet on body weight gain, apparent N and energy digestibility, food conversion efficiency, energy expenditure, nutrient oxidation and abundance of selected faecal microbiota known to be involved in non-digestible dietary fibre degradation.

## Experimental methods

### Animals and diets

Five-week-old male C57BL/6NCrl mice were individually housed in Macrolon cages Type II (Ebeco, Castrop-Rauxel, Germany) at 22°C, with a 12:12 h light–dark cycle (06.00–18.00 light). Mice were randomly assigned and fed a control diet (C; AIN-93G) for 2 weeks (adaptation phase). Subsequently, mice were switched to one of four experimental diets (*n* 10 animals per diet): C, C with 10 % inulin/oligofructose (1: 1, C + I; Orafti®HP inulin and Orafti®L95; Beneo, Mannheim, Germany), high-protein diet (HP; 40 % energy from protein) or HP diet including 10 % inulin/oligofructose (1: 1, HP + I) for a total of 4 weeks (experimental phase; Table 1). In C + I and HP + I diets, portions of starch and cellulose were substituted by inulin/FOS. Complete substitution of inulin/FOS by the non-digestible fibre cellulose only was not possible because diets were ought to be isoenergetic. However, the bomb calorimetry analysis (see later) revealed an 8⋅8 % difference in energy content between C/C + I and HP/HP + I diets. The diets were purchased from ssniff Spezialdiäten GmbH (Soest, Germany) and stored at 4°C until use. Mice had free access to water and food. Body weight and food intake were measured daily (from Monday to Friday) by the third week of the experimental phase (days (d) 15–36). The mean of daily food, dry matter (DM), crude protein, crude fat, crude fibre, carbohydrates and the carbohydrate/fat intake ratio were calculated from days 15 to 36. The daily body weight gain was calculated by subtracting the mean body weight determined on days 11 and 12 from the body weights measured during days 15–36. The food conversion efficiency was calculated as body weight gain per energy intake (g BW/MJ), based on the data from the three-week feeding period^(31)^. The carbohydrate/fat intake ratio was calculated based on the individual carbohydrate and fat intake. The experimental protocol followed was approved by the licencing authority State Office for Agriculture, Food Safety and Fishing Mecklenburg-Western Pomerania, Germany (LALLF M-V/TSD/7221.3-1-050/16).

**Table 1.** Food composition and macronutrient contents of the experimental diets

Ingredients (g/kg DM^1^)	C	C + I	HP	HP + I
Casein	200⋅0	200⋅0	454⋅0	454⋅0
Corn starch	397⋅5	337⋅5	173⋅5	103⋅5
Maltodextrin	132⋅0	122⋅0	100⋅0	100⋅0
Sucrose	100⋅0	100⋅0	100⋅0	100⋅0
Cellulose powder	50⋅0	20⋅0	50⋅0	20⋅0
L-Cysteine	3⋅0	3⋅0	5⋅0	5⋅0
Vitamin premix	10⋅0	10⋅0	10⋅0	10⋅0
Mineral premix	35⋅0	35⋅0	35⋅0	35⋅0
Choline Chloride	2⋅5	2⋅5	2⋅5	2⋅5
Inulin/FOS (1:1)^2^^,^^3^	–	100⋅0	–	100⋅0
Soyabean oil	70⋅0	70⋅0	70⋅0	70⋅0
Nutrient composition^4^ (g/kg DM)				
Crude protein	186⋅0	187⋅0	428⋅0	429⋅0
Crude fat	74⋅0	74⋅0	78⋅0	78⋅0
Crude fibre	52⋅0	120⋅0^1^	53⋅0	122⋅0^1^
Crude ash	34⋅0	34⋅0	36⋅0	36⋅0
Starch	400⋅0	341⋅0	178⋅4	107⋅0
Sucrose	117⋅0	118⋅0	120⋅0	121⋅0
Dextrin	137⋅0	127⋅0	106⋅0	106⋅0
Carbohydrate^5^/fat ratio	8⋅8	7⋅9	5⋅2	4⋅3
Measured gross energy^6^ (MJ/kg)	18⋅0	18⋅0	19⋅6	19⋅6

1 DM, dry matter.

2 Inulin/FOS calculated with 94 % crude fibre.

3 Orafti®HP inulin and Orafti®L95(Beneo).

4 Calculated nutrient composition.

5 Carbohydrates = starch + sucrose + dextrin.

6 Energy content was measured by bomb calorimetry.

### Energy expenditure and nutrient oxidation

In the fourth week of feeding the experimental diets, mice were adapted for a period of 2 h on two different days to the respiration chambers before indirect calorimetry measurements. After acclimation, mice were placed in the respiration chambers with 20 g of the experimental diets and 50 ml of water. Indirect calorimetry was performed at 22°C with 12:12 h dark–light cycle (06.00–18.00 light) to measure total energy expenditure and carbohydrate and fat oxidation as previously described^(32)^. Physical activity was recorded by an infrared motion detector converting movements into the numbers of impulses per time interval (i.e. 21 min). Data were collected using Simatic hardware and Win CC software (Siemens AG, Munich, Germany). Food and water intake were recorded over 48 h. The body weight was determined before and after gas exchange measurements to calculate the mean body weight. The daily total energy expenditure (TEE or heat production) was calculated according to Weir: TEE (kJ) = 16⋅29 × VO_2_ + 4⋅57 × VCO_2_^(33)^, where VO_2_ is the oxygen consumption (l/d) and VCO_2_ is the CO_2_ production (l/d). Net oxidation rates of fat (FOX) and carbohydrates (COX) were calculated according to Simonson and DeFronzo: FOX (g) = 1⋅69 × (VO_2_ − VCO_2_) and COX (g) = 4⋅57 × VCO_2_ − 3⋅23 × VO_2_^(34)^. The respiratory quotient (RQ) was calculated as the ratio of VCO_2_ to VO_2_, and the daily means for the 48-h measuring period were calculated. The energy balance (EB) was calculated: EB (kJ) = energy intake (kJ) − faecal energy (kJ) − TEE (kJ). One animal per group (except for C) was removed from the evaluation because of technical problems with the respiration chambers.

### Faecal and dietary N content, and gastrointestinal transit time

The faeces excreted over the 48-h period in the respiration chambers was collected and dried at 60°C for 24 h. Faecal and dietary N were determined by elemental analysis (CE-Instruments, Flash EA 1112 series, ThermoQuest, Basingstoke, UK). Apparent N digestibility was estimated by the following equation: apparent N digestibility (%) = (dietary N (g) – excreted faecal N (g)) × 100/dietary N (g)^(35)^. Three days after the indirect calorimetry experiment, mice were gavaged with 120 μl of Carmine red (10 mg/ml in drinking water)^(35)^, and the time of the expulsion of the first red faecal pellet was determined in a subset of five animals per diet.

### Calorimetric measurements

Faecal and diet samples were dried and ground. Faecal samples from two to three mice per diet were pooled yielding approximately 1 g. The energy content of the diets and faecal samples were analysed by bomb calorimetry (IKA C5003; IKA Werke, Staufen, Germany) as previously described^(36)^. The digestibility of the diet was calculated by Weitkunat *et al.*: digestibility (%) = ((dietary energy intake (kJ/2 d) – faecal energy excretion (kJ/2 d))/dietary energy intake (kJ/2 d)) × 100^(36)^.

### Quantification of faecal microbiota by RT-qPCR

Fresh faecal samples were taken in the fourth week of feeding the experimental diets and stored at −20°C until DNA isolation. Genomic DNA was extracted with 700 μl DNA extraction buffer from 70 mg frozen faeces with mechanical disruption (bead-beating for 2 × 45 s at 6500 rpm; 1⋅4 mm Precellys ceramic beats (Bertin Instruments, Montigny-le-Bretonneux, France)) using a QIAamp DNA stool mini kit (Qiagen, Hilden, Germany). DNA quality was verified on an agarose gel and the concentration was quantified using a NanoPhotometer (IMPLEM, Munich, Germany). Quantitative PCR was performed using the oligonucleotide primers (Supplementary Table S1 of Supplementary material). One PCR reaction was performed with 2 μl of diluted DNA (7⋅5 ng/μl), 0⋅5 μl of each primer (10 pmol), 6 μl 2× buffer SensiFAST SYBR No-ROX mix (Bioline, Luckenwalde, Germany) and 3 μl H_2_O PCR grade. Amplifications were detected on a Light Cycler 96 (Roche, Basel, Switzerland). One primer pair was designed to detect the bacterial 16S rRNA gene, and its amount was utilised to normalise amplicon expression^(37)^. The efficiency of amplification was calculated using LinRegPCR software, version 2014.4 (Academic Medical Centre, Amsterdam, Netherlands^(38)^, yielding efficiency values were between 1⋅75 and 1⋅85 (Supplementary Table S1 of Supplementary material). The genus *Lactobacillus* was reclassified in March 2020^(39)^. However, the primers used to detect all Lactobacillaceae classified as such until 2020. Data were quantified by qbasePlus software (Biogazelle, Gent, Belgium).

### Statistical analysis

For the study design, a power analysis was performed with CADEMO software (Windows ANOVA *F*-test version 4.03, 2000; BioMath GmbH, Rostock) to determine the minimum difference *d* according to *d* = *c_d_* * *σ*, in which the following parameters were selected: residual standard deviation *σ* 0⋅6; *c_d_* 1; type one error *α* 0⋅05 and type two error *β* 0⋅2. For each parameter, the same relative accuracy was assumed. Authors who performed statistical analyses were aware of the group allocation at all stages of the experiment. Daily measurements on the same animal were analysed by repeated measurement ANOVA using the MIXED procedure of SAS (Version 9.4, SAS Institute Inc., Cary, NC, USA). The model contained the fixed effects dietary protein level (C, HP), inulin/FOS (−I, +I), time (experimental day), and their interactions HP × I, HP × time, I × time, HP × I × time; and age and initial body weight on day 1 served as covariates. Repeated measures on the same animal were considered by the repeated statement of proc MIXED (repeated variable: time) using an autoregressive type for the block diagonal residual covariance matrix. Least-squares means (LSM) and their standard errors (se) were computed for each fixed effect in the ANOVA model. Additionally, differences of these LSM were tested using the Tukey–Kramer procedure. The SLICE statement of proc MIXED was used for performing a partitioned analysis of the LSM for the interactions HP × I, HP × time, I × time and HP × I × time. Effects of diets measured on a single time point (digestibility, faecal N excretion, physical activity and microbiota) were evaluated by ANOVA using the MIXED procedure with the fixed effect dietary protein level (C and HP), inulin/FOS (−I and +I) and age as a covariate in the model, and multiple comparisons between the dietary group levels were done using the Tukey–Kramer procedure. The results of energy metabolism (TEE, COX, FOX, RQ and EB) were tested for body weight differences using ANCOVA test^(40)^, and mean body weight during indirect calorimetry measurement was added as a covariate into the model, thereby accounting for eventual differences in body composition between groups. Spearman correlation coefficient between microbiota abundances, RQ, energy expenditure, apparent N digestibility and faecal N excretion was calculated by the CORR procedure of SAS. Results were considered statistically significant at *P* < 0⋅05.

## Results

### Body weight, body weight gain, food and energy intake

**Fig. 1.**
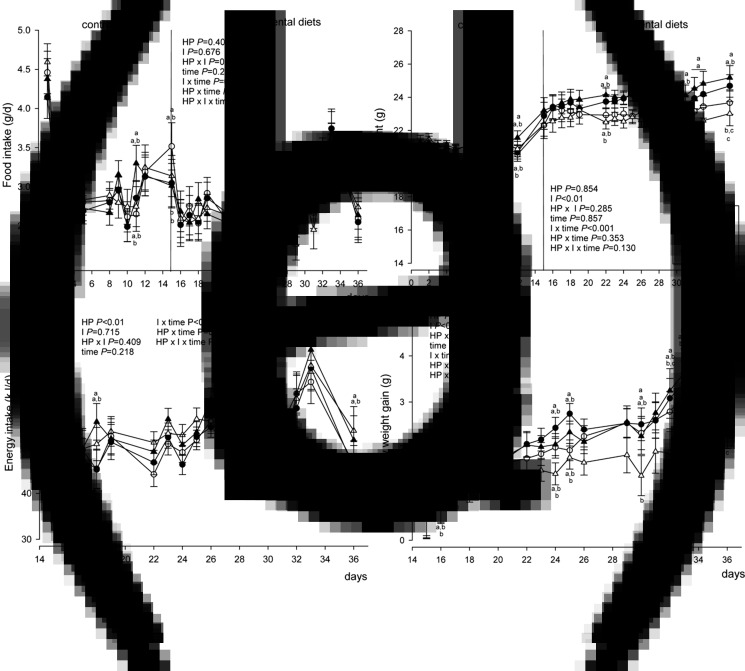
Food and energy intake, body weight and body weight gain of mice fed a control diet for 2 weeks (adaptation phase) and subsequently one of four experimental diets for 3 weeks: control ± inulin/FOS (C; C + I) or high-protein diet  ± inulin/FOS (HP; HP + I) diet. Food intake (a) and body weight (b) were recorded in the adaptation and experimental phase. Daily energy intake (c) and body weight gain (d) were calculated from days 15 to 36 of feeding experimental diets. Values are LSM and  se; *n* 10 per diet. Labelled means at one time point without a common letter differ, *P* < 0⋅05 (Tukey–Kramer test).

During the 2-week adaptation phase on the C diet, the HP + I group had a higher food intake than the C group on days 4 and 11 (Fig. 1(a)). On day 12, HP + I-fed mice had higher body weight than C-fed mice. After transfer to the experimental diets, C + I- and HP + I-fed mice reduced food and energy intake compared to C-fed mice on the first day (*P* < 0⋅05, respectively), but not during the following 3 weeks (days 15–36) of experimental diet feeding (Fig. 1(a) and (b)). From days 29 to 36, HP + I-fed mice had a significantly higher body weight than HP-fed mice (*P* < 0⋅05; Fig. 1(b)). On day 36, HP + I-fed mice showed higher body weight than C-fed mice (*P* < 0⋅05, Fig. 1(b)). Because the body weight on day 12 differed between HP + I- and C-fed mice, the body weight gain before and after the switch to one of the four experimental diets was calculated. The body weight gain in C- and C + I-fed mice did not differ, whereas C- and HP + I-fed mice gained significantly more body weight than HP-fed mice in the third week (day 32–36) on the experimental diets (*P* < 0⋅05, respectively; Fig. 1(d)). The cumulative food intake did not differ among the groups. Calculating the intake of food and macronutrients as the daily mean of days 15–36 in the experimental phase revealed that food, DM, fat and energy intake did not differ between groups (Table 2). However, carbohydrate intake was higher in both C- compared to HP-dietary groups, but lower in C + I- and HP + I- than C- and HP-fed counterparts, respectively (*P* < 0⋅05). The protein intake was higher in the HP than in C diets, irrespective of inulin/FOS content (*P* < 0⋅05, respectively; Table 2). Mice on the C + I and HP + I diets ingested 2⋅3-fold more crude fibre than mice fed C and HP, respectively (*P* < 0⋅001; Table 2). The energy intake derived from carbohydrates relative to fat was higher in HP + I- than in HP-fed mice (*P* = 0⋅007; Table 2). The food conversion efficiency was lower in HP- than in HP + I- (*P* = 0⋅019) and C + I- (*P* = 0⋅009) and C-fed mice (*P* = 0⋅022; Table 2).

**Table 2.** Average food, dry matter, macronutrient and energy intake, the carbohydrate/fat energy intake ratio and food conversion efficiency during 3 weeks feeding of control ± inulin/FOS (C; C + I) or high-protein diet ± inulin/FOS (HP; HP + I) diet.^1^ The macronutrient intake was calculated based on the dry matter content of the respective diet

Item	C	C + I	HP	HP + I	*P*-value		
					HP	I	HP × I
Cumulative food intake (g/3 weeks)	41⋅72 ± 0⋅91	42⋅19 ± 0⋅91	40⋅54 ± 1⋅02	42⋅27 ± 0⋅98	0⋅567	0⋅260	0⋅509
Food intake (g/d)	2⋅78 ± 0⋅06	2⋅81 ± 0⋅06	2⋅70 ± 0⋅07	2⋅82 ± 0⋅07	0⋅594	0⋅175	0⋅376
Dry matter intake (g/d)	2⋅41 ± 0⋅05	2⋅47 ± 0⋅05	2⋅33 ± 0⋅06	2⋅46 ± 0⋅05	0⋅422	0⋅091	0⋅486
Crude protein intake (g/d)	0⋅43 ± 0⋅02^b^	0⋅44 ± 0⋅02^b^	0⋅94 ± 0⋅02^a^	0⋅99 ± 0⋅02^a^	<0⋅001	0⋅081	0⋅228
Crude fat intake (g/d)	0⋅17 ± 0⋅00	0⋅17 ± 0⋅00	0⋅17 ± 0⋅00	0⋅18 ± 0⋅01	0⋅699	0⋅089	0⋅472
Carbohydrate intake^2^ (g/d)	1⋅51 ± 0⋅02^a^	1⋅38 ± 0⋅02^b^	0⋅88 ± 0⋅03^c^	0⋅77 ± 0⋅03^d^	<0⋅001	<0⋅001	0⋅759
Carbohydrate/fat intake ratio	8⋅80 ± 0⋅004^a^	7⋅87 ± 0⋅004^b^	5⋅18 ± 0⋅004^c^	4⋅27 ± 0⋅004^d^	<0⋅001	<0⋅001	<0⋅01
Crude fibre intake (g/d)	0⋅12 ± 0⋅01^b^	0⋅28 ± 0⋅01^a^	0⋅12 ± 0⋅01^b^	0⋅28 ± 0⋅01^a^	0⋅725	<0⋅001	0⋅717
Daily energy intake (kJ/d)	50⋅20 ± 1⋅12^b^	50⋅76 ± 1⋅12^b^	52⋅90 ± 1⋅24^a,b^	55⋅68 ± 1⋅12^a^	<0⋅01	0⋅166	0⋅349
Total energy intake (kJ/3 weeks)	751⋅0 ± 17⋅4^b^	794⋅3 ± 19⋅2^a,b^	759⋅4 ± 17⋅4^a,b^	828⋅3 ± 18⋅5^b^	<0⋅01	0⋅252	0⋅482
Carbohydrate/fat energy ratio	4⋅16 ± 0⋅10^c^	4⋅31 ± 0⋅10^b,c^	4⋅74 ± 0⋅11^b^	5⋅30 ± 0⋅11^a^	<0⋅001	<0⋅01	0⋅057
Food conversion efficiency (g BW/MJ)	4⋅08 ± 0⋅30^a^	4⋅24 ± 0⋅30^a^	2⋅60 ± 0⋅33^b^	4⋅09 ± 0⋅32^a^	<0⋅05	<0⋅05	<0⋅05

1 Values are LSM and se; *n* 10 per dietary group.

2 Carbohydrates = starch + sucrose + dextrin.

Labelled means in a row without a common letter differ, *P* < 0⋅05 (Tukey–Kramer test).

### Energy metabolism and physical activity

**Fig. 2.**
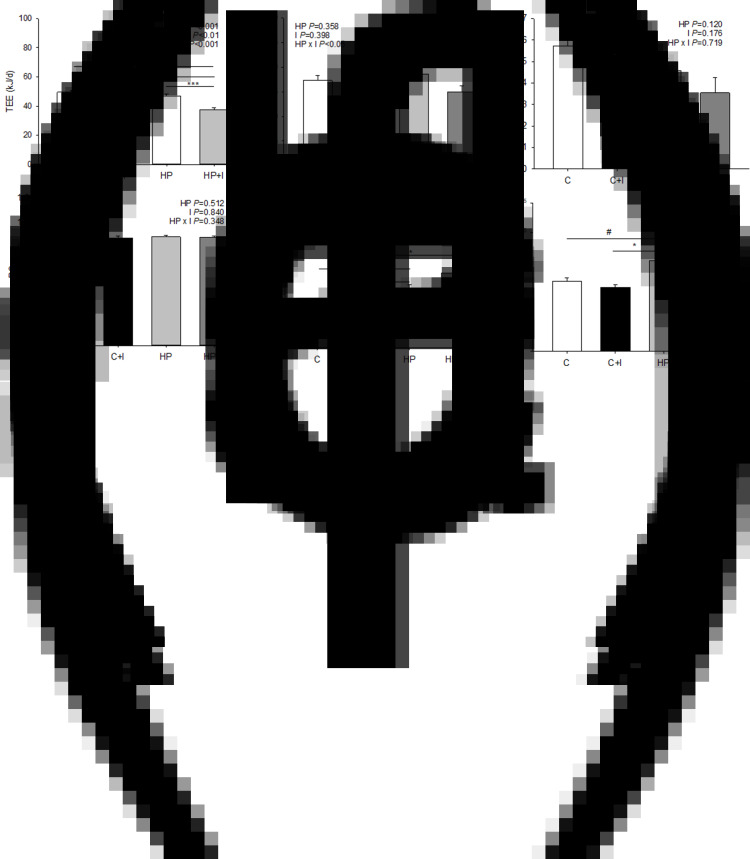
Components of energy expenditure in mice fed a control ± inulin/FOS (C; C + I) or a high-protein diet ± inulin/FOS (HP; HP + I) diet for 3 weeks. The gas exchange was analysed for 48 h in respiration chambers. Daily total energy expenditure (TEE) (a), carbohydrate oxidation (COX) (b) and fat oxidation (FOX) (c), each normalised to metabolic body weight (mBW), respiratory quotient (RQ) (d), energy balance (EB) (e) and physical activity (f). Values are LSM and se; C *n* 10; C + I, HF, HF + I, HP, HP + I *n* 9 per diet. #0⋅06 < *P* < 0⋅1, *P < 0⋅05, **P < 0⋅01, ***P < 0⋅001 (Tukey–Kramer test).

The TEE was lower in mice fed the HP + I than HP, C + I and C (*P* < 0⋅001; Fig. 2(a)). Similarly, COX tended to be lower in HP + I- compared to HP-fed mice (*P* = 0⋅1; Fig. 2(b)). In contrast, FOX and RQ were comparable among the groups during the 48-h measuring period (Fig. 2(c) and (d)). The EB was higher in HP + I- than in C- (*P* = 0⋅01) and C + I-fed mice (*P* = 0⋅02); Fig. 2(e)). The EB of HP-fed mice tended to be lower in C- and C + I-fed mice (*P* = 0⋅09, respectively), but the physical activity was greater in mice fed the HP than in HP + I, C and C + I (*P* < 0⋅05; Fig. 2(f)). During the stay in the respiration chambers, the mean body weight was higher in mice fed inulin/FOS-containing diets (*P* < 0⋅05, Supplementary Fig. S1(a) of Supplementary material), but the food and energy intakes were comparable among dietary groups (Supplementary Fig. S1(b) and (c) of Supplementary material). The 48-h water intake was 1⋅5–1⋅6-fold higher in HP- than in C-groups independent of inulin/FOS content (*P* < 0⋅05; Supplementary Fig. S1(d) of Supplementary material).

### Faecal N excretion, apparent N digestibility and gastrointestinal transit time

The amount of faecal excreta and faecal DM were lower in C + I- and HP + I- compared to those of in C- (*P* < 0⋅05) and HP-fed mice (*P* < 0⋅001; Table 3). The water loss via faeces tended to be 83 % higher in HP- compared to that of in HP + I-fed mice (*P* = 0⋅09; Table 3). The 48-h dietary N intake was lower in C- than in HP-fed mice, independently of inulin/FOS (*P* < 0⋅001; Table 3). The 48-h faecal N excretion was higher in mice fed the HP compared to that of in C (*P* < 0⋅001), C + I (*P* < 0⋅01) and HP + I (*P* < 0⋅01) diets. The faecal N excretion/intake ratio was significantly lower in C- than in C + I-fed mice (*P* = 0⋅02), but did not differ between HP- and HP + I-fed mice, while it was lower in mice of both HP than of both C-groups (*P* = 0⋅01). Furthermore, mice fed a C diet had a higher apparent N digestibility than C + I-fed mice (*P* < 0⋅04), while it was not different between HP- and HP + I-fed mice. However, the apparent N digestibility was higher in HP + I- than in C + I-fed mice (*P* < 0⋅001; Table 3). Inulin/FOS and the dietary protein content had no effect on the gastrointestinal transit time (Table 3). The faecal energy content was significantly higher in mice fed inulin-containing than inulin-free diets (*P* < 0⋅05), while the faecal energy excretion was not different among the dietary groups. In addition, diet digestibility was significantly higher in HP + I- than in C-, C + I- and HP-fed mice (*P* < 0⋅01, respectively) and significantly higher in C + I- than in C-fed mice (*P* = 0⋅02; Table 3).

**Table 3.** Faecal characteristics and excretions, nitrogen (N) intake, apparent N digestibility, gastrointestinal transit time and diet digestibility in mice fed a control ± inulin/FOS (C; C + I) or a high-protein diet ± inulin/FOS (HP; HP + I) diet for 3 weeks. Data and samples were collected during the 48-h indirect calorimetry measurements^1^

Item	C	C + I	HP	HP + I	HP	*P*-value	
						I	HP × I
Faecal excretion (g/2 d)	0⋅44 ± 0⋅02^a^	0⋅36 ± 0⋅02^b^	0⋅51 ± 0⋅02^a^	0⋅35 ± 0⋅02^b^	0⋅125	<0⋅001	0⋅075
Faecal dry mass excretion (g/2 d)	0⋅41 ± 0⋅02^a^	0⋅33 ± 0⋅02^b,c^	0⋅46 ± 0⋅02^a^	0⋅32 ± 0⋅02^c^	0⋅208	<0⋅001	0⋅107
Faecal water (%)	7⋅8 ± 0⋅8	7⋅8 ± 0⋅8	9⋅9 ± 0⋅9	8⋅5 ± 0⋅9	0⋅132	0⋅367	0⋅436
Faecal water excretion (mg/2 d)	35⋅5 ± 4⋅8^AB^	27⋅7 ± 5⋅2^AB^	49⋅1 ± 5⋅7^A^	29⋅3 ± 5⋅5^B^	0⋅182	<0⋅05	0⋅274
Faecal energy content^2^ (kJ/g)	13⋅8 ± 0⋅1^b^	14⋅8 ± 0⋅1^a^	13⋅9 ± 0⋅1^b^	14⋅5 ± 0⋅1^a^	0⋅378	<0⋅001	0⋅096
Faecal energy excretion (kJ/2 d)	11⋅2 ± 0⋅8	10⋅9 ± 0⋅8	13⋅1 ± 0⋅8	11⋅9 ± 0⋅8	0⋅081	0⋅359	0⋅604
Faecal C/N ratio	8⋅5 ± 1⋅2	10⋅5 ± 1⋅2	8⋅0 ± 1⋅3	10⋅5 ± 1⋅3	0⋅830	0⋅088	0⋅780
Faecal N excretion (mg/2 d)	15⋅5 ± 1⋅9^b^	18⋅0 ± 1⋅9^b^	27⋅9 ± 2⋅1^a^	19⋅2 ± 2⋅0^b^	<0⋅01	0⋅134	<0⋅05
N intake (mg/2 d)	171⋅0 ± 15⋅6^b^	145⋅7 ± 15⋅6^b^	426⋅3 ± 15⋅6^a^	390⋅1 ± 15⋅6^a^	<0⋅001	<0⋅05	0⋅449
Faecal N excretion/N intake ratio	0⋅09 ± 0⋅01^b^	0⋅13 ± 0⋅01^a^	0⋅06 ± 0⋅01^c,d^	0⋅05 ± 0⋅01^d^	<0⋅01	<0⋅05	0⋅135
Apparent N digestibility (%)	81⋅5 ± 1⋅5^b^	75⋅1 ± 1⋅6^c^	86⋅4 ± 1⋅7^a,b^	89⋅6 ± 1⋅7^a^	<0⋅001	0⋅335	<0⋅01
Gastrointestinal transit time (min)	367 ± 66	331 ± 66	298 ± 68	316 ± 68	0⋅531	0⋅892	0⋅698
Diet digestibility (%)	94⋅3 ± 0⋅1^c^	95⋅0 ± 0⋅2^b^	94⋅4 ± 0⋅1^b,c^	95⋅9 ± 0⋅2^a^	<0⋅01	<0⋅001	<0⋅05

1 Values are LSM and se; *n* 10 per dietary group, with the exception of transit time were *n* 5 per dietary group.

2 Faecal samples from *n* 3–5 mice per diet were pooled for the analysis of faecal energy content by bomb calorimetry.

Labelled means in a row without a common lower case letter differ, *P* < 0⋅05; labelled means in a row without a common upper case letter differ, *P* < 0⋅1 (Tukey–Kramer test).

### Faecal microbiota abundance

**Fig. 3.**
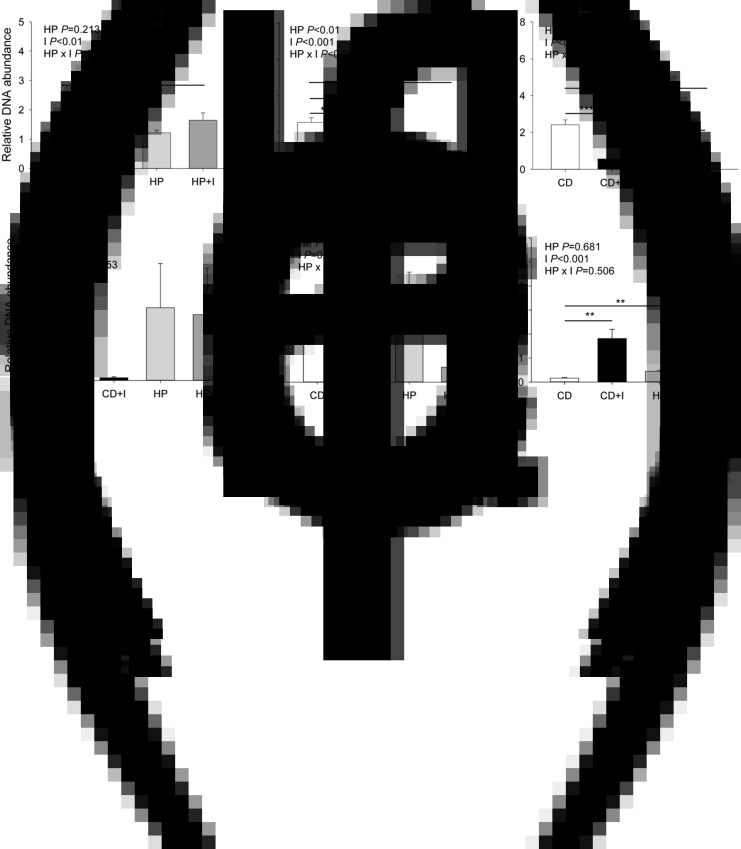
Faecal microbiota groups in mice fed a control ± inulin/FOS (C; C + I) or a high-protein diet ± inulin/FOS (HP; HP + I) diet for 3 weeks. (a) *Bacteroides–Prevotella* group, (b) *Clostridium coccoides* group, (c) *Clostridium leptum*, (d) *Lactobacillus*, (e) *Enterobacteriaceae* and (f) the ratio of *Bacteroides–Prevotella* group to total *Clostridium* abundance. Values are LSM and se; *n* 10 per diet. *P < 0⋅05, **P < 0⋅01, ***P < 0⋅001 (Tukey–Kramer test).

The investigation of the faecal microbiota revealed that the DNA abundance of the *Bacteroides–Prevotella* group was 2⋅3-fold higher in faeces of mice fed the C + I and HP + I than in C diets (*P* < 0⋅05; Fig. 3(a)). Faeces in the C group had a 2⋅3–5⋅1-fold higher *C. coccoides* group DNA abundance than faeces from C + I-, HP- and HP + I-groups (*P* < 0⋅001; Fig. 3(b)). The faecal *C. leptum* DNA abundance was 4⋅3–6⋅5-fold higher in C- than in C + I- and HP + I-fed mice (*P* < 0⋅001; Fig. 3(c)). The HP + I-fed mice had 79 % lower faecal *C. leptum* DNA abundance than HP-fed mice (*P* < 0⋅001; Fig. 3(c)). No significant difference in the relative DNA abundance of faecal *Lactobacillus* and *Enterobacteriaceae* was found among the experimental diets (Fig. 3(d) and (e)). The ratio of the *Bacteroides–Prevotella* group to the total *Clostridium* abundance was 3⋅8–10⋅7-fold higher in inulin/FOS-supplemented compared to C- and HP-fed groups (*P* < 0⋅01, Fig. 3(f)). Besides, mice fed the HP diet showed a 2⋅7-fold higher ratio between the *Bacteroides–Prevotella* group and total *Clostridium* abundance than C-fed mice (*P* < 0⋅01). We found significant inverse correlations between the abundances of the *Bacteroides–Prevotella* group and *C. leptum* and the *C. coccoides* group, respectively (Supplementary Table S2 of Supplementary material). The abundance of the *C. coccoides* group was positively correlated with *C. leptum* and *Enterobacteriaceae*, respectively. Furthermore, there was a positive correlation between *Lactobacillus* and *Enterobacteriaceae* abundances.

### Correlation between microbiota abundances, Respiratory Quotient, energy expenditure, apparent N digestibility and faecal N excretion

A positive correlation was found between *C. leptum* abundance and TEE (Table 4). *Lactobacillus* abundances correlated inversely with TEE, but directly with energy intake and apparent N digestibility.

**Table 4.** Spearman correlation coefficients between the abundance of faecal microbial groups, energy expenditure, nutrient oxidation, energy intake, faecal nitrogen (N) excretion and apparent N digestibility

Item	BacPrev	ClosCos	ClosLep	Lacto	Entero
TEE	−0⋅291	0⋅109	0⋅352*	−0⋅371*	−0⋅013
COX	−0⋅111	0⋅011	0⋅193	−0⋅088	−0⋅057
FOX	−0⋅105	0⋅127	0⋅105	−0⋅068	0⋅103
RQ	0⋅042	−0⋅044	0⋅048	0⋅011	−0⋅088
Energy intake	0⋅039	−0⋅043	−0⋅093	0⋅397*	−0⋅027
Faecal N excretion	−0⋅028	−0⋅041	0⋅118	0⋅152	−0⋅073
Apparent N digestibility	0⋅205	0⋅004	−0⋅150	0⋅548*	0⋅095

BacPrev, *Bacteroides–Prevotella* group; ClosCos, *Clostridium coccoides* group; ClosLep, *Clostridium leptum*; Lacto, *Lactobacillus*; Entero, *Enterobacteriaceae*.

* *P* < 0⋅05.

## Discussion

In contrast to our expectations, 3-week feeding of an HP diet containing inulin/FOS increased body weight gain in C57BL/6 mice above the level of C-fed mice, while feeding the HP diet alone reduced body weight gain relative to HP + I- and C-fed mice. This could not be explained by differences in energy intake, which did not differ among the groups. Mice in the HP + I compared to those in the HP group consumed a greater proportion of fat relative to carbohydrates, which corresponds to a greater portion of energy intake from fat. We and others have reported earlier that HP diets can reduce food intake and body weight in normal-weight mice^(17,41–43)^. However, we did not observe an effect of inulin/FOS included in the C diet on food intake and body weight gain. Our finding is in line with previous experiments in BALB/c mice in which no effects on body weight were observed when a control diet was supplemented with 10 % of different fermentable dietary fibres, such as inulin, FOS, XOS, galacto-oligosaccharide (GOS), apple pectin, polydextrose or beta-glucan for 3 weeks of feeding^(44)^. Likewise in rats, feeding a 55 % HP diet containing 10 % FOS for 5 weeks had no effect on body weight and fat mass compared to controls albeit the energy intake was 10 % lower in the HP diet with FOS than in the control diet^(45)^. In contrast, rats fed with a high-fat diet (40 %) containing 10 % inulin for 3 weeks decreased cumulative food intake but not body weight and fat mass^(46)^. Based on these findings, it can be concluded that the dietary protein and fat levels exert a major influence on inulin/FOS's effect on body weight gain.

In order to elucidate the potential underlying mechanisms, we investigated the energy expenditure, carbohydrate and fat oxidation and energy balance over a 48-h time period after 3 weeks on the experimental diets. In HP + I-fed mice, the 15 % lower carbohydrate intake resulted in a 17 % lower carbohydrate-to-fat intake ratio which is reflected by the 19 % difference in COX between HP + I- and HP-fed mice. The 42-fold higher energy balance (EB) in HP + I- than in C- and C + I-fed mice pointed to a strong impact of the lower TEE on EB in the HP + I group. Although C- and C + I-fed mice still gained body weight in week 4 on the experimental diet, C- and C + I-fed mice were in slightly negative EB during the 2 d of indirect calorimetry measurement, which was due to lower food intake in the respiration chamber. Of note, the body weight change (before and after indirect calorimetry measurement) was not altered among the dietary groups. However, inulin/FOS in the C diet had no impact on TEE, EB, physical activity, respiratory quotient (RQ), carbohydrate and fat oxidation when compared to C. A previous study revealed that feeding a 60 % HP diet to C57BL/6 mice for 12 weeks reduced TEE compared to mice fed a control diet^(17)^, which, however, was not apparent in HP- and C-fed mice in our study. This is likely due to the higher dietary protein content used in the earlier study^(17)^. However, as indicated by the lower TEE, HP + I-fed mice dissipated less dietary energy as heat, had lower physical activity, and gained more body mass than HP-fed mice, suggesting that mice fed the HP + I were more effective in converting dietary energy into body mass as underlined by the higher diet digestibility and food conversion efficiency. It seems that the higher starch content of the HP diet provides more glucose which is preferably oxidised, while, inulin/FOS in the HP + I diet, which can be completely fermented in the colon and caecum, deliver more short-chain fatty acids (SCFA). For example, SCFA infused in the caecum of mice are substrates for glucose, cholesterol and lipid synthesis^(47)^, but if this mechanism is entirely responsible to deposit more body mass in HP + I-fed mice remains to be investigated. In addition, it remains unclear if the higher body mass gain of HP + I-fed mice is a result of an increase in body fat or muscle mass. Unfortunately, we did not measure body composition in our study, but recently, it has been reported that rats fed with a 55 % high-protein diet with and without 10 % FOS for 5 weeks did not differ in body composition, even compared to the control group^(45)^.

The question arose, if energy metabolism is related to gut microbiota. Therefore, we performed correlation analyses between faecal gut microbiota abundances and energy expenditure, RQ and nutrient oxidation. Interestingly, TEE was positively correlated with the abundance of *C. leptum* and negatively correlated with *Lactobacillus*, but not with COX. In contrast to our finding, feeding a whey-inulin compared to a whey-cellulose diet to obesity-prone (OPCD) rats reduced the copy number of *Lactobacillus* and energy expenditure^(48)^, pointing to a direct correlation between *Lactobacillus* and energy expenditure. Conclusively, the negative correlation between TEE and the abundance of *Lactobacillus* in the present study is mainly driven by the dietary protein but not by inulin/FOS content.

Mice fed the HP diet showed higher physical activity than C-, C + I- and HP + I-fed mice. Earlier studies reported no significant increase in physical activity in mice in response to HP relative to C feeding^(17,49)^. The discrepancy between earlier^(17,49)^ and our results may be due to the difference in feeding duration (3 weeks *v*. 12 weeks) or dietary protein concentration (40 % *v*. 60 % or 47⋅9 % protein), whereas the mouse strain was the same in all studies. The greater water intake with HP than C feeding is in concordance with an earlier study, demonstrating that 60 % HP diet feeding for 12 weeks led to 75 % higher water intake than C diet feeding in C57BL/6 mice^(17)^. The higher water intake with the HP diet is due to the increase in protein degradation and the need for greater urinary urea excretion^(50)^, independently of the inulin/FOS content in our study.

High-protein diets are incompletely digested accompanied by high faecal N excretion^(51)^. Our present results reveal that the inclusion of inulin/FOS in an HP diet reduces the amount of faecal N excretion without altering the transit time of the digest. In contrast to our findings, the addition of 7⋅5 % FOS or XOS in place of starch increased faecal N excretion while it reduced renal excretion of N by 20–30 % compared to fibre-free control diet in rats^(8)^. Adult dogs receiving a diet containing 1 % FOS for 3 weeks showed unaltered urinary N excretion and unaltered nitrogen balance compared to those with the control diet^(52)^. Fewer studies focused on N intake and N balance while feeding an HP diet. Feeding HP diets supplemented with inulin (12⋅5 g/kg DM) increased faecal N excretion and decreased the ratio of urinary to faecal N output compared with inulin-free diet feeding of finishing pigs^(51)^. However, the 30 % lower faecal N excretion on the HP + I compared to the HP diet in the present study might be partly related to the higher apparent N digestibility, while the ratio of faecal N excretion/N intake was not different between HP + I- and HP-fed mice. We show here that mice on the HP diet had higher faecal water content than with HP + I feeding, while the faecal water content of C-fed mice was comparable to C + I- and HP + I-fed mice. The reason for the higher faecal water content with HP feeding but not with the inclusion of inulin/FOS may be linked to the increase of undigested protein reaching the colon and increasing proteolytic fermentation^(53)^. The proteolytic fermentation favours higher osmotic pressure and therefore greater water transfer into the intestinal lumen^(53)^. Inulin and FOS do not seem to induce higher faecal water excretion as shown in our study. Comparably, Pinna *et al.* found no differences in faecal water content when adult dogs received 1⋅5 g FOS/kg DM to a low- or high-protein diet for 28 d^(53)^.

In the large intestine, a vast range of bacterial species grow and are specialised for a particular ecological niche^(54)^. Prebiotics and amino acids represent the main energy substrate for bacteria promoting microbial growth^(2,27,28,35)^. While there has been an extensive focus on the bifidogenic effect of inulin^(2,55,56)^, only few reports describe the inulin/FOS effect on other bacteria species. Species belonging to the genus *Bacteroides* are versatile polysaccharide- and FOS-fermenters^(2,7)^. Our present findings show a higher faecal DNA abundance of the Gram-negative *Bacteroides–Prevotella* group after feeding C + I and HP + I than C. Feeding a high-fibre diet containing 21⋅6 % inulin/FOS (1:1) until the age of 25 weeks led to higher *Bacteroides–Prevotella* DNA abundances than in rats fed a control diet^(57)^. Furthermore, saccharolytic gut bacteria belong to the *Clostridium* or *Lactobacillus* genus^(2)^. Our findings of reduced *C. coccoides* and *C. leptum* DNA abundances in faeces of HP + I- and C + I-fed mice are in accordance with the results of previous studies describing reduced *C. coccoides* and *C. leptum* DNA abundances in the faeces of rats after feeding with a high-fibre diet with 21⋅6 % inulin/FOS until 25 weeks of age compared to C or HP diet feeding^(57)^. A higher *Bacteroides–Prevotella* to total *Clostridium* ratio was visible in both inulin/FOS groups reflecting a shift in the microbiota composition by prebiotics independent of the dietary background. Our relative quantification of *Lactobacillus* and *Enterobacteriaceae* did not reveal differences related to inulin/FOS of either an HP or a C diet.

Additional analysis revealed a moderately positive correlation between *Lactobacillus* DNA abundance, apparent N digestibility and energy intake. This effect is again likely due to the different dietary protein levels, because an increase of protein in the diet has been shown to increase the abundance of Lactobacillaceae^(58)^. In contrast to our findings, feeding of an HP + I diet (200 g protein and 12⋅5 g inulin/kg DM) to finishing pigs for 3 weeks did not alter apparent N digestibility, *Lactobacilli* spp. abundances in the colon^(51)^.

Although genders may differently respond to the same diet^(59)^, the present study investigated the effect of inulin/FOS in a high-protein diet only in male mice, and thus potential sex differences between diets need to be investigated in future studies. Another aspect is the dosage of inulin/FOS fed to mice, which translates to 43–44 g inulin/FOS/day for a 60–90 kg adult man. Such amount can be critical, as inulin dosages >15 g/d can induce undesirable side effects, e.g. flatulence, abdominal pain and bloating^(12,13)^. Levels ≥ 20 g of inulin/FOS per day have been shown to induce cramps and abdominal pain in humans^(60)^.

Another limitation of the present study is that we measured only a selected and limited number of microbiota candidates known from the literature to be responsive to prebiotics. In the future, a microbiome study should be performed to elucidate further species involved in the adaptation to HP + I feeding.

In summary, our results demonstrate that, in contrast to our hypothesis, the inclusion of 10 % inulin/FOS to a 40 % HP diet did not reduce body weight gain as compared to the HP diet. Rather, HP + I feeding increased digestibility and body weight gain, but reduced total energy expenditure, physical activity, faecal N excretion and *C. leptum* DNA abundance compared to HP diet feeding. Thus, feeding an HP diet containing inulin/FOS altered the intermediary metabolism and mildly affected the gut microbiota. Our data indicate that mice fed the HP + I diet converted nutrients into body mass more effectively than counterparts fed the HP diet alone and achieved comparable body weight gain as mice fed the C diet. However, the inclusion of inulin/FOS to the C diet did not alter body weight gain, nutrient utilisation efficiency and energy expenditure, indicating that the combination of the dietary protein level and inulin/FOS is the major driver of the metabolic changes observed.

## Supplementary material

For supplementary material accompanying this paper visit http://dx.doi.org/10.1017/jns.2021.42. click here to view supplementary material
