# Supplementary material for: A high-protein diet containing inulin/oligofructose supports body weight gain associated with lower energy expenditure and carbohydrate oxidation, and alters faecal microbiota in C57BL/6 mice
Source: J Nutr Sci. 2021 Jul 13;10:e50. doi: 10.1017/jns.2021.42 (PMC8278163; doi:10.1017/jns.2021.42)
Supplement: Supplementary file 1 [file S2048679021000422sup001.docx]

**A high-protein diet supplemented with inulin/FOS supports body weight gain associated with lower energy expenditure, carbohydrate oxidation and alters fecal microbiota in C57BL/6 mice**

Franziska Koch, Michael Derno, Martina Langhammer, Armin Tuchscherer, Harald M. Hammon, Manfred Mielenz, Cornelia C. Metges, Björn Kuhla

**Online Supplementary Material**

**Supplemental Table 1.** Primers used for relative bacterial DNA quantification in fecal samples.

| Targeted Bacterial Groups and Genera | Primers | bp | PCR efficiency | Ref. Number |
| --- | --- | --- | --- | --- |
| Bacteroides - | for 5′- CCTWCGATGGATAGGGGTT-3′ | 131 | 1.85 | ^(1)^ |
| Prevotella group | rev 5′- CACGCTACTTGGCTGGTTCAG-3′ |  |  |  |
| Clostridium | for 5′-GCTTCTTAGTCARGTACCG-3′ | 139 | 1.81 | ^(2)^ |
| coccoides group | rev 5′- ACTCCTACGGGAGGCAGC-3′ |  |  |  |
| Clostridium leptum | for 5′-GAATTAAACCACATACTCCACTGCTT-3′ | 116 | 1.83 | ^(1)^ |
| group | rev 5′- CCTTCCGTGCCGSAGTTA-3′ |  |  |  |
| Enterobacteriaceae | for 5′-CATTGACGTTACCCGCAGAAGAAGC-3′ | 190 | 1.75 | ^(3)^ |
|  | rev 5′- CTCTACGAGACTCAAGCTTGC-3′ |  |  |  |
| Lactobacillus | for 5′-TCCTACGGGAGGCAGCAGT-3′ | 32 | 1.83 | ^(4)^ |
|  | rev 5′- TGG AAG ATT CCC TAC TGC-3′ |  |  |  |
| Total bacteria | for 5′- ACTCCTACGGGAGGCAG -3′ | 199 | 1.85 | ^(5)^ |
|  | rev 5′- GTATTACCGCGGCTGCTG -3′ |  |  |  |

**Supplemental Table 2.** Spearman correlation coefficients between the abundances of specific fecal bacterial groups.

| Item | BacPrev | ClosCos | ClosLep | Lacto | Entero |
| --- | --- | --- | --- | --- | --- |
| Bacteroides – Prevotella group | - | - | - | - | - |
| Clostridium coccoides group | -0.538*** | - | - | - | - |
| Clostridium leptum | -0.485** | 0.799*** | - | - | - |
| Lactobacillus | 0.010 | 0.265 | 0.207 | - | - |
| Enterobacteriacea | -0.311 | 0.474** | 0.300 | 0.475** | - |

* *P* < 0.05; ** *P* < 0.01; *** *P* < 0.001.

**Supplemental Fig. 1.** Mean body weight (a), food intake (b),energy intake (c) and water intake (d) of mice fed a control +/- inulin/FOS (C; C+I) or a high-protein diet +/- inulin/FOS (HP; HP+I) during the 48-h measuring period in the respiration chamber. Values are LSmeans ± SE. C n=10; C+I, HF, HF+I, HP, HP+I, each n=9. * *P* < 0.05 (Tukey-Kramer test).

C
C+I
HP
HP+I

**Supplemental Fig. 2.** Continuous (a) carbohydrate oxidation (COX) and (b) fat oxidation (FOX) during the 48-h gas exchange measurement. Single time points are calculated as LSmean of mice fed C +/- inulin/FOS (C, C+I) or HP +/- inulin/FOS (HP, HP+I) diets. C n=10; C+I, HF, HF+I, HP, HP+I, each n=9.

**References**

1. Do TT, Hindlet P, Waligora-Dupriet AJ *et al.* (2014) Disturbed intestinal nitrogen homeostasis in a mouse model of high-fat diet-induced obesity and glucose intolerance. *American journal of physiology Endocrinology and metabolism* **306**, E668-680.

2. Louie TJ, Cannon K, Byrne B *et al.* (2012) Fidaxomicin preserves the intestinal microbiome during and after treatment of Clostridium difficile infection (CDI) and reduces both toxin reexpression and recurrence of CDI. *Clinical infectious diseases : an official publication of the Infectious Diseases Society of America* **55 Suppl 2**, S132-142.

3. Bartosch S, Fite A, Macfarlane GT *et al.* (2004) Characterization of bacterial communities in feces from healthy elderly volunteers and hospitalized elderly patients by using real-time PCR and effects of antibiotic treatment on the fecal microbiota. *Applied and environmental microbiology* **70**, 3575-3581.

4. Hallam MC, Barile D, Meyrand M *et al.* (2014) Maternal high-protein or high-prebiotic-fiber diets affect maternal milk composition and gut microbiota in rat dams and their offspring. *Obesity* **22**, 2344-2351.

5. Anitha M, Reichardt F, Tabatabavakili S *et al.* (2016) Intestinal dysbiosis contributes to the delayed gastrointestinal transit in high-fat diet fed mice. *Cellular and molecular gastroenterology and hepatology* **2**, 328-339.
